# Supplementary material for: Novel Driver Strength Index highlights important cancer genes in TCGA PanCanAtlas patients
Source: PeerJ. 2022 Aug 11;10:e13860. doi: 10.7717/peerj.13860 (PMC9375969; doi:10.7717/peerj.13860)
Supplement: Supplemental Information 2 [file peerj-10-13860-s002.zip › COHORTS/genes plots/stage/2021_8_16_14_9_distribution_stage_males_IV.pdf]

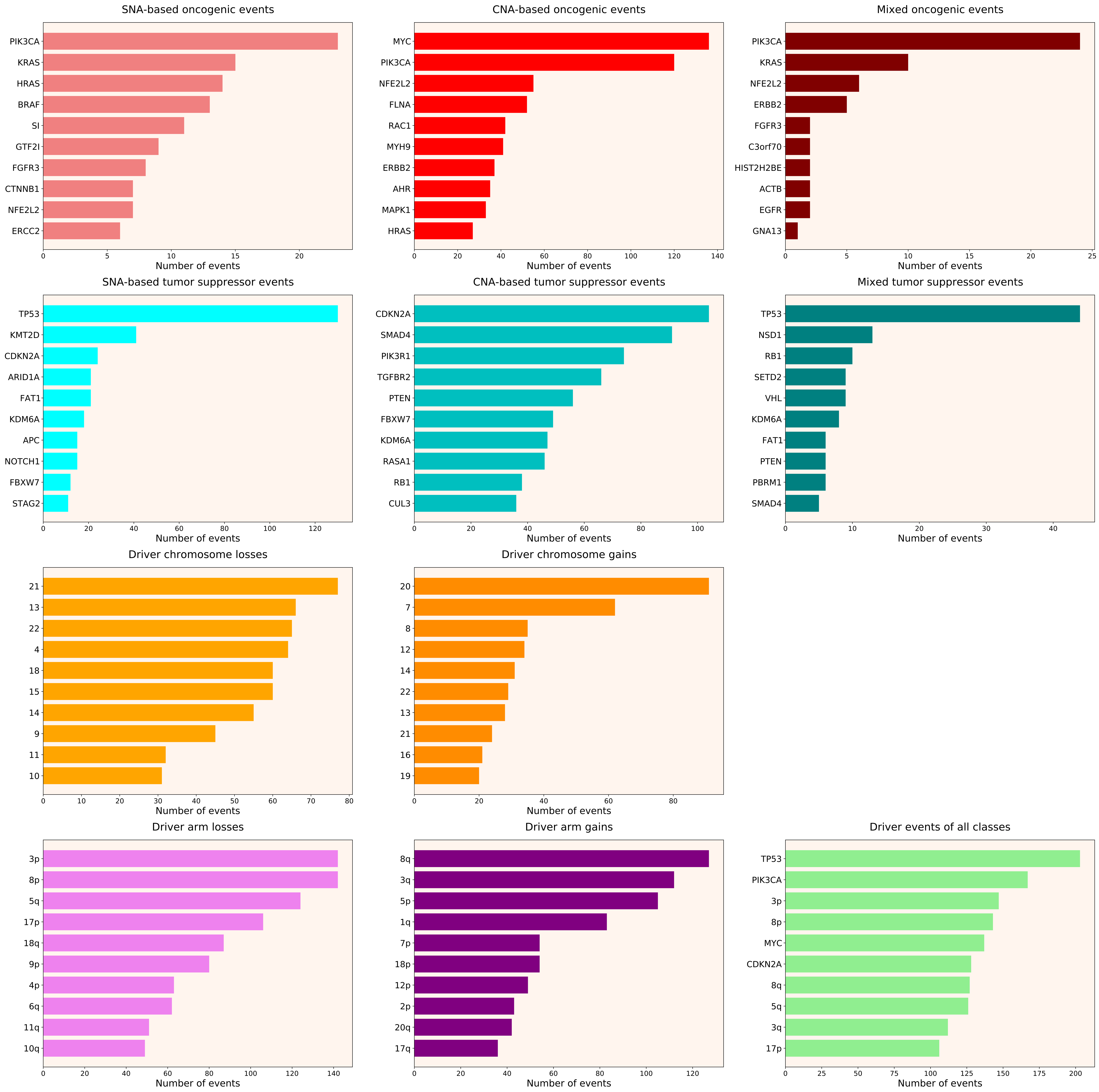
SNA-based oncogenic events
PIK3CA
KRAS
HRAS
BRAF
SI
GTF2I
FGFR3
CTNNB1
NFE2L2
ERCC2
0 5 10 15 20
Number of events
CNA-based oncogenic events
MYC
PIK3CA
NFE2L2
FLNA
RAC1
MYH9
ERBB2
AHR
MAPK1
HRAS
0 20 40 60 80 100 120 140
Number of events
Mixed oncogenic events
PIK3CA
KRAS
NFE2L2
ERBB2
FGFR3
C3orf70
HIST2H2BE
ACTB
EGFR
GNA13
0 5 10 15 20 25
Number of events
SNA-based tumor suppressor events
TP53
KMT2D
CDKN2A
ARID1A
FAT1
KDM6A
APC
NOTCH1
FBXW7
STAG2
0 20 40 60 80 100 120
Number of events
CNA-based tumor suppressor events
CDKN2A
SMAD4
PIK3R1
TGFBR2
PTEN
FBXW7
KDM6A
RASA1
RB1
CUL3
0 20 40 60 80 100
Number of events
Mixed tumor suppressor events
TP53
NSD1
RB1
SETD2
VHL
KDM6A
FAT1
PTEN
PBRM1
SMAD4
0 10 20 30 40
Number of events
Driver chromosome losses
21
13
22
4
18
15
14
9
11
10
0 10 20 30 40 50 60 70 80
Number of events
Driver chromosome gains
20
7
8
12
14
22
13
21
16
19
0 20 40 60 80
Number of events
Driver arm losses
3p
8p
5q
17p
18q
9p
4p
6q
11q
10q
0 20 40 60 80 100 120 140
Number of events
Driver arm gains
8q
3q
5p
1q
7p
18p
12p
2p
20q
17q
0 20 40 60 80 100 120
Number of events
Driver events of all classes
TP53
PIK3CA
3p
8p
MYC
CDKN2A
8q
5q
3q
17p
0 25 50 75 100 125 150 175 200
Number of events
